# Long Noncoding RNA MALAT1 and Colorectal Cancer: A Propensity Score Analysis of Two Prospective Cohorts

**DOI:** 10.3389/fonc.2022.824767

**Published:** 2022-04-26

**Authors:** Heng Li, Yuxue Zhang, Yanlong Liu, Zhangyi Qu, Yupeng Liu, Jiping Qi

**Affiliations:** ^1^ Department of Pathology, The First Affiliated Hospital of Harbin Medical University, Harbin, China; ^2^ Department of Hygiene Microbiology, Public Health School of Harbin Medical University, Harbin, China; ^3^ Department of Colorectal Surgery, The Third Affiliated Cancer Hospital of Harbin Medical University, Harbin, China; ^4^ Department of Epidemiology and Biostatistics, School of Public Health and Management, Wenzhou Medical University, Wenzhou, China

**Keywords:** colorectal neoplasms, MALAT1, prognosis value, cohort, propensity score analysis (PSA)

## Abstract

**Background:**

Previous researches have shown that the aberrant expression of Metastasis associated in lung adenocarcinoma transcript 1 (MALAT1) in tumour tissues may serve as a biomarker for colorectal cancer (CRC) prognosis. However, these previous studies have small sample sizes and lacked validation from independent external populations. We therefore aimed to clarify the prognostic value of MALAT1 expression status in CRC patients using a large cohort and validate the findings with another large external cohort.

**Methods:**

The prognostic association between MALAT1 expression status and CRC outcomes was evaluated initially in a prospective cohort in China (n=164) and then validated in an external TCGA population (n=596). In the initial cohort, MALAT1 expression levels were quantified by quantitative reverse transcriptase polymerase chain reaction. Propensity score (PS) adjustment method was used to control potential confounding biases. The prognostic significance was reported as PS-adjusted hazard ratio (HR) and corresponding 95% confidence interval (CI).

**Results:**

There was no statistically significant association between MALAT1 expression status and CRC patient overall survival (OS) or disease free survival (DFS) in both initial cohort and external validation cohort populations. When combining these populations together, the results did not change materially. The summarized HR_PS-adjusted_ were 1.010 (95% CI, 0.752-1.355, *P*=0.950) and 1.170 (95% CI, 0.910-1.502, *P*=0.220) for OS and DFS, respectively.

**Conclusions:**

MALAT1 expression status is not associated with prognostic outcomes of CRC patients. However, additional larger population studies are needed to further validate these findings.

## Introduction

Colorectal cancer (CRC) is a leading cause of cancer-related death worldwide. It is the second- and third- most commonly diagnosed cancer in females and males, respectively, and more than 1.93 million newly diagnosed CRC patients and 935,173 deaths were estimated to occur in 2020 worldwide (1). By 2035, the incidence and mortality of CRC are predicted to increase to 2.5 million new cases and 1.1 million deaths (2, 3). Unlike most developed countries, the incidence of CRC in China has increased significantly in both men and women from 2000 to 2011 (4). In 2020, approximately 555,477 new cases and 286,162 deaths were estimated to occur in China, which accounted for about 30% of all annually diagnosed CRC cases and CRC-related deaths worldwide (1).

Even though mortality from CRC has significantly decreased over the past two decades, the 5-year relative survival rate is about 64% in the United States, and the rate remains less than 50% in low-income regions (3, 5). Approximately half of these new cases spread as micrometastases at the time of initial diagnosis. Consequently, about 45% of patients suffer from recurrence or metastases after lesion resection (6). To date, the pathological tumour staging system and specific histological characteristics are the most common prognostic predictors for CRC patients after surgery. However, patients with similar clinical/pathological features often experience different clinical outcomes (7). Therefore, an effectual predictive biomarker is urgently needed for the prediction of disease outcomes. In our previous studies, we developed a series of blood-based DNA methylation biomarkers for CRC prognosis (8, 9). Long noncoding RNAs are becoming hotspots in the research field of tumour biomarkers. Metastasis associated in lung adenocarcinoma transcript 1 (MALAT1) is a well-studied lncRNA (10). MALAT1, which is firstly found in a study of early-stage non-small-cell lung cancer, is 8.5 kb in length and is located at 11q13 (11). Previous studies have revealed that MALAT1 is commonly upregulated in human cancer tissues of diverse organ origins and that MALAT1 induces proliferation, migration and invasion of cancer cells *in vitro* and tumor metastasis in mice (12–16). Subsequent mechanistic studies have demonstrated a vital function of MALAT1 in the development and progression of various cancers, including CRC (17–19). However, the results remain ambivalent (20, 21).

Recently, several studies have shown that the aberrant expression of MALAT1 in tumour tissues may serve as a biomarker for CRC prognosis (22–27). However, these previous studies had small sample sizes. None of these studies validated their results in external populations. In order to clarify whether the expression status of MALAT1 in tumour tissues is associated with CRC prognosis or the clinical characteristics of CRC patients, we performed this prospective cohort analysis with a relatively large sample size and a long-term follow-up period. We further used the datasets of colon and rectum adenocarcinoma from The Cancer Genome Atlas (TCGA) as an independent external cohort population to validate our findings.

## Materials and Methods

### Patient Samples and Inclusion Criteria

**Figure 1:**
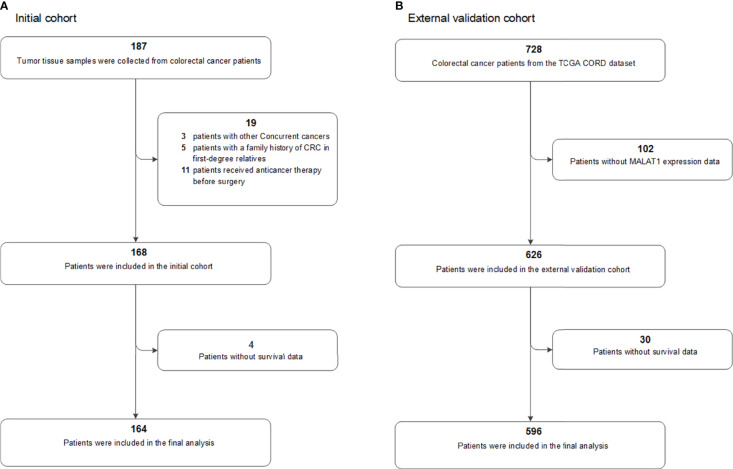
Flow chart of participants included and analysed in the **(A)** initial and **(B)** external validation cohorts. COAD, colon adenocarcinoma; CRC, colorectal cancer; TCGA, The Cancer Genome Atlas.

This study was approved by the Harbin Medical University Ethics Review Board (Harbin, China). The study design and patient selection strategy have been published previously (8, 9). All of the patients provided written informed consent. The inclusion and exclusion criteria were as follows: (1) all patients were newly diagnosed with stage I-IV primary CRC, and their diagnosis was histologically confirmed by a senior pathologist (HL); (2) fresh-frozen tumour tissues were collected from all patients; (3) patients with other cocurrent cancers were excluded (n=3); (4) patients with a family history of CRC in first-degree relatives were excluded (n=5); and (5) patients who received anticancer therapy before surgery were excluded (n=11). Four cases lacked follow-up data and were excluded from this analysis. Finally, a total of 164 patients in our initial prospective cohort of CRC patients were included in the final prognostic analysis (**Figure 1**).

All CRC patients were diagnosed and operated on at the First Affiliated Hospital and the Third Affiliated Hospital of Harbin Medical University between May 2010 and December 2012. The tumour specimens were staged according to the 2009 seventh version of the AJCC TNM staging system. Their clinical characteristics and medical records were collected. The primary outcome was overall survival (OS), defined as the time from surgery to death from any cause. The secondary outcome was disease-free survival (DFS), defined as the time from surgery to local or regional relapse, distant metastasis, or CRC-specific death, whichever came first. Outcomes were observed during the follow-up period through March 15, 2018 *via* an established protocol. Postoperative patients were followed up at 3-6 month intervals for the first year and then annually. We used a telephone-delivered follow-up questionnaire to collect information on the date and cause of death of CRC patients. The recorded date and cause of death of each CRC patient were validated using the medical certification of death and the Harbin Death Registration system. Among these 164 eligible CRC patients, the median follow-up period was 61.1 months (ranging from 4.9 to 80.8 months), and 75 patients died.

### RNA Extraction and qRT-PCR Assays

Fresh tumour tissue samples were collected and immediately stored at -80°C. Total RNA was extracted from fresh frozen tissues (0.5 g) using TRIzol reagent (Invitrogen). cDNA was reverse transcribed from 2 μg total RNA using MultiScribe™ reverse transcriptase (Applied Biosystems). The RNA and cDNA concentrations were measured using NanoDrop 2000c (Thermo Fisher, USA). cDNA was then amplified and quantified by quantitative reverse transcriptase polymerase chain reaction (qRT-PCR) with Fast SYBR^®^ Green Master Mix (Applied Biosystems) on the LightCycler 480 platform (Roche).The housekeeping gene GAPDH was selected as an internal control. A no-template control was included in each batch and all reactions were performed in triplicate. The primer sequences are as follows. MALAT1 (NR_002819.4): F-(5’- GCTCTGTGGTGTGGGATTGA -3’), MALAT1-R-(5’- GTGGCAAAATGGCGGACTTT -3’); GAPDH (NM_002046.7): F-(5’-GGTGGTCTCCTCTGACTTCAACA -3’), R-(5’- CCAAATTCGTTGTCATACCAGGAAATG -3’). Melting curve analysis was used to monitor the specificity of PCR reactions. The resulting data were analysed using the Gene Scanning and TM Calling modules (Roche). Two coauthors (HL and YXZ) blinded to the outcomes independently recorded the results. The relative expression level of MALAT1 was determined using the 2^-ΔCt^ method. The ΔCt value of each sample was calculated by subtracting the average Ct value of MALAT1 from the average Ct value of GAPDH. According to the median value of 2^-ΔCt^, the patients were categorized into higher or lower MALAT1 expression groups.

### External Validation Dataset

The colorectal dataset (CORD) from TCGA was used as an external validation population. The MALAT1 expression profile data, clinicopathologic information, and survival data were downloaded from the TCGA database and the UCSC Xena resource (28, 29). After excluding those without MALAT1 expression data (n=102) or survival data (n=30), a total of 596 patients were included in our analyses (**Figure 1**), including 475 patients with colon cancer and 121 with rectal cancer. The median follow-up period for these 596 patients was 22.5 months, with a range of 0.2 to 150.1 months, and a total of 121 patients died.

The gene expression RNA-seq-HTSeq-FPKM-UQ dataset for TCGA colon and rectum adenocarcinoma was performed using the UCSC Xena website tools and then used in our analyses. The relative quantification of MALAT1 expression level is presented as N-fold differences and termed ‘N_Malat1_’, which was determined by dividing the value of MALAT1 expression by the value of GAPDH. Then, the patients were categorised into the higher (≥median of N_Malat1_) or lower (< median of N_Malat1_) groups.

### Statistical Analysis

We used a Cox proportional hazards regression model to calculate the sample size. Given a pre-estimated overall survival rate of 50% in this initial cohort population, a sample size of 128 cases was required to achieve 90% power to detect an estimated hazard ratio (HR) of 1.5 with a two-sided 5% level of statistical significance. Finally, we included additionally 25% more patients and targeted a total sample size of 164 patients. The sample size was estimated using PASS software (version 11.0.7, NCSS LLC., USA).

We reported means (standard deviations) and counts (frequencies) for continuous and categorical variables, respectively. To minimize the covariate differences between groups, we performed a PS-based analysis (30). Group differences were compared using the standardised differences method with a significant imbalance level of standardised difference ≥25%. The PS value was calculated with the MALAT1 expression level as the dependent variable using a multivariate logistic regression model that included demographic factors and clinical/pathological characteristics. We used the PS-adjustment method in order to incorporate all of the patients in our analysis (31).

Survival curves were estimated by the Kaplan-Meier method, and the survival rate differences between groups were examined with log-rank tests. Univariate and PS-adjusted multivariate Cox proportional hazards regression models were used to assess the prognostic significance, and the results are reported as hazard ratios (HRs) and 95% confidence intervals (CIs). The associations between MALAT1 expression status and the clinical/pathological covariates are reported as odds ratios (ORs) and 95% CIs. Statistical significance was defined as a two-sided P < 0.05. All statistical analyses were conducted with SPSS Statistics (v.23.0, IBM, USA).

### Sensitivity Analysis

Several predesigned sensitivity analyses were performed to explore the robustness of the results. Firstly, we compared the univariate HR and the PS-adjusted HR using the confounding RR (32), which was calculated to evaluate the relative impact of the PS adjustment on the results. Secondly, we performed a conventional multivariate Cox regression analysis as a sensitivity analysis. Additionally, for the external cohort population, we performed a *post hoc* sensitivity analysis by excluding those patients with a shorter follow-up duration (≤ 1 or ≤ 3 months) in order to explore the potential confounding impact. Finally, we performed extensive *post hoc* subgroup analysis according to the clinical/pathological factors. In the *post hoc* subgroup analyses, we used the Bonferroni adjustment method to correct for the level of statistical significance.

### Meta Analysis

In order to better understand the current evidence for the association between MALAT1 expression and CRC prognosis, we systematically reviewed the relevant researches and performed a meta-analysis. We systematically searched for eligible studies assessing the prognostic significance of MALAT1 expression on CRC patient outcomes in PubMed, EmBase, and ProQuest through May 25, 2020. The inclusion criteria were as follows: (1) prospective cohort studies addressing the prognostic associations of MALAT1 and CRC outcomes; (2) studies that reported effect estimates including HRs with corresponding CIs; (3) studies with a sample size of more than 50 participants; and (4) there was no restriction on language, race, or any other participant characteristics. Data extraction was conducted independently by two coauthors (HL and YXZ). The maximally adjusted effect sizes and 95% CIs were extracted and summarized using random-effects models. The Q test and the I^2^ statistic were used to test the between-study heterogeneity. The pooled effect estimates are presented as forest plots. We performed E-value analysis (33), as a *post-hoc* sensitivity analysis, to explore whether an unmeasured confounding factor could explain the observed associations.

## Results

### MALAT1 and Patient Outcomes

In the initial cohort, we analysed the MALAT1 expression levels in a series of 164 tumour tissues from primary CRC patients with known clinical/pathological status and long-period follow-up outcomes. After PS adjustment, all of these covariates between groups reached a balance (Standardised mean difference < 0.25, **Table S1** in the **Supplementary Materials**). There was no prognostic association between MALAT1 expression status and the CRC patient outcomes. The univariate HRs were 1.428 (95% CI, 0.901-2.263, P=0.130) and 1.525 (95% CI, 0.975-2.387, P=0.065) for OS and DFS, respectively. After PS adjustment, the HR_PS-adjusted_ were 1.087 (95% CI, 0.657-1.797, P=0.745) and 1.150 (95% CI, 0.709-1.865, P=0.570) for OS and DFS, respectively. Subgroup analyses by the clinical/pathological factors showed similar results (**Table 1**).

**Table 1:** Prognostic associations of MALAT1 expression and colorectal cancer outcomes in the initial population.

Factors and Subgroups	No. of Patients	MALAT1 Expression Level		Overall Survival		Disease Free Survival	
		Lower (ref.)	Higher	PS-Adjusted HR (95% CI)	P-value	PS-Adjusted HR (95% CI)	P-value
**Overall**	164	82	82	1.087 (0.657-1.797)	0.745	1.150 (0.710-1.865)	0.570
**Gender**							
Female	93	51	42	1.133 (0.590-2.173)	0.708	1.328 (0.689-2.559)	0.396
Male	71	31	40	0.987 (0.448-2.174)	0.974	0.965 (0.477-1.953)	0.921
**Age (yr)**							
< 60	90	46	44	1.108 (0.559-2.199)	0.769	1.153 (0.592-2.246)	0.675
≥ 60	74	36	38	1.025 (0.488-2.153)	0.948	1.138 (0.568-2.279)	0.716
**BMI (kg/m2)**							
Normal weight	87	44	43	1.268 (0.621-2.589)	0.514	1.150 (0.581-2.279)	0.688
Overweight or Obese	77	38	39	0.824 (0.397-1.711)	0.604	1.026 (0.509-2.066)	0.943
**Tumor Location**							
Right Colon	19	10	9	1.866 (0.407-8.547)	0.422	0.911 (0.204-4.069)	0.902
Left Colon	44	19	25	1.046 (0.322-3.395)	0.940	1.074 (0.341-3.378)	0.903
Rectum	101	53	48	1.115 (0.610-2.038)	0.725	1.332 (0.748-2.372)	0.330
**AJCC Stage**							
1	16	10	6	0.002 (0.000-40.664)	0.391	0.002 (0.000-40.664)	0.391
2	66	26	40	0.707 (0.331-1.510)	0.370	0.859 (0.420-1.755)	0.676
3	66	35	31	1.702 (0.705-4.113)	0.237	1.790 (0.782-4.097)	0.168
4	16	11	5	1.037 (0.297-3.623)	0.954	2.399 (0.672-8.565)	0.178
**CEA (ng/mL)**							
≤ 5	83	45	38	0.677 (0.293-1.562)	0.360	0.682 (0.296-1.572)	0.369
> 5	81	37	44	1.394 (0.717-2.711)	0.328	1.528 (0.811-2.876)	0.189
**History of Cancers**							
No	142	69	73	1.195 (0.688-2.077)	0.527	1.256 (0.740-2.133)	0.398
Yes	22	13	9	0.676 (0.183-2.493)	0.577	0.766 (0.233-2.629)	0.671
**CA19-9 (U/mL)**							
≤ 37	122	68	54	1.208 (0.620-2.353)	0.578	1.484 (0.768-2.868)	0.240
> 37	42	14	28	0.547 (0.259-1.157)	0.114	0.427 (0.207-0.880)	0.021
**Tumor Size (mm)**							
≤ 40	58	31	27	2.086 (0.896-4.859)	0.088	2.063 (0.915-4.651)	0.081
> 40	106	51	55	0.788 (0.419-1.484)	0.461	0.857 (0.471-1.560)	0.614
**Histopathological Morphology**							
Protruding	114	60	54	0.971 (0.494-1.908)	0.931	1.085 (0.561-2.100)	0.808
Infiltrating ulcer	50	22	28	1.336 (0.625-2.587)	0.454	1.373 (0.668-2.822)	0.389
**Differentiation**							
Low to Medium	103	52	51	1.525 (0.836-2.780)	0.169	1.650 (0.916-2.974)	0.095
High	61	30	31	0.568 (0.224-1.440)	0.233	0.590 (0.247-1.410)	0.235
**Adjuvant Chemotherapy**							
No	102	54	48	0.774 (0.379-1.579)	0.482	0.808 (0.415-1.575)	0.531
Yes	62	28	34	1.748 (0.839-3.643)	0.136	1.843 (0.888-3.825)	0.101

The results from the initial cohort study were validated by using a large external cohort involving 596 CRC patients from TCGA. After PS adjustment, all of these clinical/pathological covariates between the groups were balanced (**Table S2**). These findings were consistent with the results from the initial cohort. The univariate HRs were 1.072 (95% CI, 0.750-1.532, P=0.703) and 1.266 (95% CI, 0.948-1.690, P=0.110) for OS and DFS, respectively. After PS adjustment, the HR_PS-adjusted_ were 0.971 (95% CI, 0.676-1.396, P=0.876) and 1.177 (95% CI, 0.878-1.577, P=0.276) for OS and DFS, respectively. Subgroup analyses showed similar results (**Table 2**).

**Table 2:** Prognostic associations of MALAT1 expression and colorectal cancer outcomes in the external validation population.

Factors and Subgroups	No. of Patients	MALAT1 Expression Level		Overall Survival		Disease Free Survival	
		Lower (ref.)	Higher	PS-Adjusted HR (95% CI)	P-value	PS-Adjusted HR (95% CI)	P-value
**Overall**	596	298	298	0.971 (0.676-1.396)	0.876	1.177 (0.878-1.577)	0.276
**Gender**							
Female	274	137	137	1.103 (0.646-1.881)	0.720	1.290 (0.835-1.994)	0.252
Male	322	161	161	0.839 (0.508-1.383)	0.491	1.087 (0.729-1.619)	0.683
**Age (yr)**							
< 60	174	92	82	1.680 (0.713-3.960)	0.235	1.513 (0.835-2.743)	0.172
≥ 60	422	206	216	0.880 (0.587-1.319)	0.536	1.099 (0.783-1.542)	0.585
**BMI (kg/m^2^)**							
Normal weight	195	116	79	1.195 (0.654-2.186)	0.562	1.450 (0.869-2.421)	0.155
Overweight or Obese	401	182	219	0.987 (0.627-1.555)	0.956	1.136 (0.794-1.625)	0.486
**Tumor Location**							
Right Colon	250	130	120	0.856 (0.514-1.426)	0.551	0.896 (0.574-1.397)	0.627
Left Colon	225	111	114	1.232 (0.645-2.352)	0.528	1.477 (0.907-2.406)	0.117
Rectum	121	57	64	0.867 (0.348-2.160)	0.759	1.354 (0.673-2.727)	0.396
**AJCC Stage**							
1	106	57	49	0.942 (0.149-5.970)	0.950	1.836 (0.553-6.100)	0.321
2	222	113	109	0.445 (0.213-0.926)	0.030	0.792 (0.467-1.344)	0.388
3	179	92	87	1.144 (0.618-2.116)	0.669	1.211 (0.727-2.015)	0.462
4	89	36	53	1.082 (0.545-2.150)	0.822	1.342 (0.756-2.382)	0.314
**CEA (ng/mL)**							
≤ 5	263	143	120	0.837 (0.419-1.672)	0.615	1.139 (0.677-1.915)	0.623
> 5	333	155	178	1.037 (0.675-1.594)	0.869	1.172 (0.821-1.673)	0.381
**History of polyps**							
No	407	212	195	1.027 (0.671-1.571)	0.903	1.126 (0.800-1.586)	0.497
Yes	189	86	103	0.814 (0.403-1.647)	0.568	1.301 (0.734-2.307)	0.368

We then combined the PS-adjusted HRs from the initial and external populations together by using random effect models, and found no prognostic significance of MALAT1 expression status in the CRC patient outcomes. The pooled HR_PS-adjusted_ were 1.010 (95% CI, 0.752-1.355, P=0.950) and 1.170 (95% CI, 0.910-1.502, P=0.220) for OS and DFS, respectively. The pooled effect estimates for the subgroup populations also showed similar results (**Table 3**).

**Table 3:** Prognostic associations of MALAT1 expression and colorectal cancer outcomes in the combined populations.

Factors and Subgroups	No. of Patients	MALAT1 Expression Level		Overall Survival		Disease Free Survival	
		Lower (ref.)	Higher	PS-Adjusted HR (95% CI)	P-value	PS-Adjusted HR (95% CI)	P-value
**Overall**	760	380	380	1.010 (0.752-1.355)	0.950	1.170 (0.910-1.502)	0.220
**Gender**							
Female	367	188	179	1.115 (0.737-1.685)	0.606	1.301 (0.905-1.871)	0.155
Male	393	192	201	0.879 (0.576-1.341)	0.549	1.056 (0.746-1.494)	0.759
**Age (yr)**							
< 60	264	138	126	1.303 (0.763-2.225)	0.332	1.341 (0.861-2.091)	0.195
≥ 60	496	242	254	0.911 (0.639-1.300)	0.609	1.106 (0.816-1.500)	0.515
**BMI (kg/m^2^)**							
Normal weight	282	160	122	1.225 (0.773-1.942)	0.388	1.334 (0.886-2.010)	0.168
Overweight or Obese	478	220	258	0.939 (0.638-1.381)	0.748	1.112 (0.809-1.530)	0.513
**Tumor Location**							
Right Colon	269	140	129	0.926 (0.571-1.503)	0.756	0.897 (0.586-1.373)	0.616
Left Colon	269	130	139	1.186 (0.673-2.091)	0.556	1.407 (0.898-2.204)	0.136
Rectum	222	110	112	1.033 (0.625-1.708)	0.900	1.341 (0.859-2.093)	0.196
**AJCC Stage**							
1	122	67	55	0.928 (0.147-5.879)	0.937	1.824 (0.549-6.059)	0.326
2	288	139	149	0.556 (0.328-0.943)	0.029	0.815 (0.533-1.247)	0.346
3	245	127	118	1.303 (0.786-2.157)	0.304	1.348 (0.873-2.080)	0.178
4	105	47	58	1.071 (0.587-1.956)	0.822	1.481 (0.878-2.498)	0.141
**CEA (ng/mL)**							
≤ 5	346	188	158	0.768 (0.451-1.309)	0.332	0.982 (0.623-1.549)	0.939
> 5	414	192	222	1.131 (0.789-1.624)	0.503	1.249 (0.916-1.703)	0.160

### Sensitivity Analysis

The results of the confounding RR analyses are shown in **Table S3**. Overall, the confounding RRs demonstrated no significant change after PS adjustment. However, all of these confounding RRs were smaller than 1, suggesting that the PS adjustment generated more conservative effect estimates. Another sensitivity analysis using conventional multivariate Cox regression models found very similar results (**Table S4**). For the external cohort validation analysis, a sensitivity analysis excluding fourteen patients with follow-up periods of no more than 1 month or 3 months did not materially change the results (**Table S5**).

### Meta-Analysis of MALAT1 and Patient Outcomes

**Figure 2:**
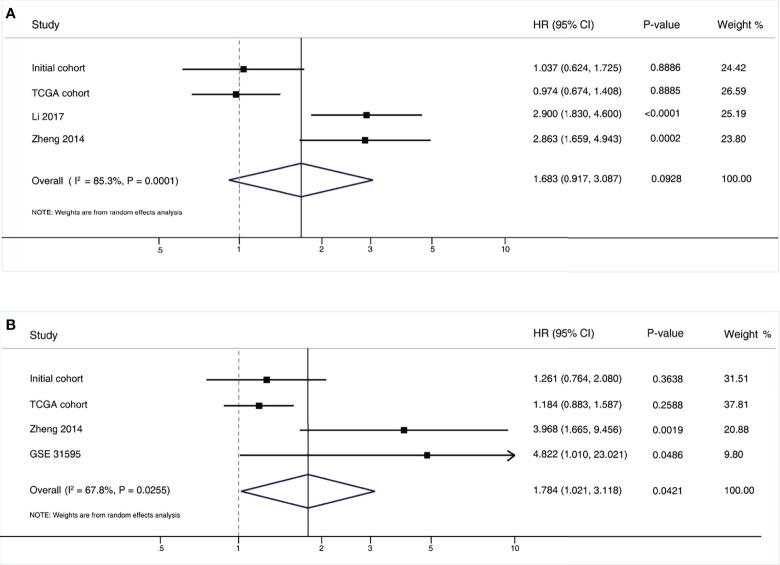
Pooled results of the meta-analysis for MALAT1 expression and colorectal cancer outcomes. **(A)** Overall survival; **(B)** Disease free survival. CI, confidence interval; HR, hazard ratio.

To further assess the robustness of the results, we performed a systematic meta-analysis. The pooled results are shown in **Figure 2**. Briefly, three additional eligible studies were included in this meta-analysis. By pooling these results together, we still did not find a positive prognostic association for OS, with a summarized HR of 1.683 (95% CI, 0.917-3.087; P=0.093). For DFS, there was a marginally positive association between higher MALAT1 expression and worse DFS, with a summarized HR of 1.784 (95% CI, 1.021-3.118; P=0.042).

### MALAT1 and Clinical/Pathological Characteristics

We sought associations between the MALAT1 expression level and the clinical/pathological characteristics in CRC patients (**Tables S6** and **S7**). We found a significantly positive association between MALAT1 overexpression and higher CA19-9 levels (P=0.016), and higher T stage (P=0.030) in the initial cohort population. In the external cohort, a significantly strong association between MALAT1 overexpression and overweight or obese (≥ 25 kg/m2) was observed (P=0.001). For the other clinical/pathological factors, there was no positive relationship.

## Discussion

In our initial cohort, there was no prognostic association between MALAT1 expression status and CRC patient outcomes. This finding was confirmed in the external TCGA cohort. Furthermore, the consistency among extensive sensitivity analyses proved the robustness of the results. To our best knowledge, the present study is the largest population cohort addressing the prognostic effect of MALAT1 on CRC patient outcomes. We initially performed a prospective cohort analysis with a long-term follow-up period of 7 years. Then we used the CORD patient cohort from TCGA as external validation datasets. No association of MALAT1 expression status with the OS or DFS of the CRC patients was found in our analysis, which was inconsistent with the findings from several previous studies.

A total of eight relevant studies assessed the association of MALAT1 expression with the CRC prognosis (17–19, 22, 23, 25–27). Most of these studies reported that CRC patients with higher MALAT1 expression in tumour tissues had worse clinical outcomes with a shorter OS or DFS. However, the sample sizes of these previous studies were all small, ranging from 30 to 146 cases. In addition, univariate Cox hazard ratio regression models were used in most of these studies, and only Zheng (22) and Li (23) took the potential impact of multifactor confounders into consideration. None of these studies conducted PS-based analyses. The PS-based method is a powerful statistical tool to control for confounding bias and is often more practical and statistically more efficient than conventional strategies of multivariate statistical analyses (30, 31), and it has been increasingly used to reduce the impact of confounders in observational studies, especially studies with small sample size.

Based on the inclusion criteria, three eligible studies were finally included in the meta-analysis. The pooled results supported the notion that there was no association between MALAT1 expression and the OS of CRC patients. For DFS, a marginally positive prognostic significance was observed; however, the E-values of both the point estimate and the lower CI limit of the pooled results were small, suggesting that a hypothetical residual confounding factor would fully explain the observed association for DFS (**Table S8**). Future large population cohorts are needed to further validate this issue for DFS. Given the rigorousness and better performance of controlling confounders with the PS methods used in this study, conclusions were drawn mainly according to the findings from our initial and external validation populations.

Subgroup analyses by AJCC stage revealed a marginally better OS in stage II CRC patients with higher MALAT1 expression than those with lower expression. The HR_PS-adjusted_ was 0.556 (95% CI, 0.328-0.943) with a P-value of 0.029, which did not reach statistical significance according to the Bonferroni correction method (α = 0.0125). In the CA19-9 higher level subgroup, CRC patients with higher MALAT1 expression had a longer OS than those with lower expression. However, this finding cannot be validated in the TCGA external cohort population, due to the lack of eligible data. Therefore, the findings from subgroup analyses should be interpreted with caution.

Recent studies have reported that MALAT1 exhibits both oncogenic and tumor suppressive functions (20, 34). Initially, MALAT1 was identified as a prognostic factor for early-stage non-small cell lung cancer metastasis (11); subsequent studies found that MALAT1overexpression was associated with poor outcomes in various cancers (35). In contrast, several studies have reported significantly lower MALAT1 expression in metastatic than primary breast cancer tumor tissue and have reported a positive correlation between MALAT1 overexpression and better patient survival, suggesting a tumor suppressive role of MALAT1 in CRC and breast cancer (21, 36, 37). Our present study, as the largest sample size study to date, with external validation from an independent population, showed that MALAT1 expression was not associated with prognosis in CRC patients and that MALAT1 may have a potential tumor suppressive role in certain subpopulations. Most recently, MALAT1-based therapeutic approaches have gained increasing interest (38, 39). Current researches involving MALAT1-targeted therapies are still in early stages and have not yet entered clinical testing (40). Due to the dual function of MALAT1 (oncogenicity or tumor suppression), the development of MALAT1-targeted therapies should be addressed with caution (20, 40).

This study had several major strengths, including the novel PS-based analysis, a relatively large population, validation by using an external cohort population, and validation by meta-analysis. However, our present study had certain limitations. First, confounding bias was the major limitation due to the nature of the observational cohort study design. The findings of confounding RR analyses suggested that those confounders could overstate the prognostic association of MALAT1 with CRC patient outcomes. In a conservative manner, we used the PS-adjustment method to maximally control for the impact of potential confounders on the results. It is known that the PS method is a powerful statistical tool to reduce the likelihood of confounding bias in observational studies. Another limitation is the lack of detailed information about adjuvant chemotherapy from both our initial cohort and the external cohort.

## Conclusions

On the basis of our assessments of internal and external validity, the precision of the effect estimates, and the consistency of the results from various subgroup analyses and extensive sensitivity analyses, we concluded that MALAT1 expression status is not associated with prognostic outcomes of CRC patients. However, additional larger population studies are needed to further validate these findings.

## Data Availability Statement

The original contributions presented in the study are included in the article/**Supplementary Material**. Further inquiries can be directed to the corresponding authors.

## Ethics Statement 

The studies involving human participants were reviewed and approved by the Medical Ethics Committee of Harbin Medical University. The patients/participants provided their written informed consent to participate in this study.

## Author Contributions

HL and YZ contributed equally to this work. JQ had full access to all of the data in this work and take responsibility for the integrity of the data and the accuracy of the data analysis. YPL and JQ contributed to study conception and design. YPL and JQ were responsible for study supervision. YPL contributed to funding acquisition. HL, YLL, and JQ contributed to sample collection. HL, YZ, and YPL contributed to RNA preparation, RT-PCR experiments, and acquisition and assembly of data. HL, YZ, YLL, and YPL contributed to analysis and interpretation of data. YZ, HL, and YPL contributed to writing the original draft. YLL, ZQ, and JQ contributed to revise this manuscript critically for important intellectual content. All authors contributed to the review and final approval of this manuscript.

## Funding

This research was funded by the Natural Science Foundation of Heilongjiang Province (grant number YQ2019H021 to YPL); the China Postdoctoral Science Foundation (grant number 2018M641875 to YPL); and the Wenzhou Science & Technology Bureau Scientific Research Project (grant number Y20190191 to YPL).

## Conflict of Interest

The authors declare that the research was conducted in the absence of any commercial or financial relationships that could be construed as a potential conflict of interest.

## Publisher’s Note

All claims expressed in this article are solely those of the authors and do not necessarily represent those of their affiliated organizations, or those of the publisher, the editors and the reviewers. Any product that may be evaluated in this article, or claim that may be made by its manufacturer, is not guaranteed or endorsed by the publisher.

## Supplementary Material

Supplementary Table 1 Detailed balance before and after propensity score adjustment in the initial cohort population.

Supplementary Table 2 Detailed balance before and after propensity score adjustment in the external TCGA cohort population.

Supplementary Table 3 Sensitivity analysis by using confounding RR analysis.

Supplementary Table 4 Sensitivity analysis by using of conventional multivariate Cox hazard regression method.

Supplementary Table 5 Sensitivity analysis by excluding patients with shorter follow-up duration.

Supplementary Table 6 Associations between MALAT1 expression level and clinical/pathological characteristics in the initial cohort population.

Supplementary Table 7 Associations between MALAT1 expression level and clinical/pathological characteristics in the external cohort population.

Supplementary Table 8 E-value analyses for the pooled effect estimates of the meta-analysis.

## Abbreviations

CI, confidence interval; CORD, colorectal dataset; CRC, colorectal cancer; DFS, disease free survival; HR, hazard ratio; MALAT1, Metastasis associated in lung adenocarcinoma transcript 1; OS, overall survival; PS, propensity score; TCGA, The Cancer Genome Atlas.
