# Supplementary material for: Long Noncoding RNA MALAT1 and Colorectal Cancer: A Propensity Score Analysis of Two Prospective Cohorts
Source: Front Oncol. 2022 Apr 26;12:824767. doi: 10.3389/fonc.2022.824767 (PMC9088002; doi:10.3389/fonc.2022.824767)
Supplement: Supplementary Table 1 — Detailed balance before and after propensity score adjustment in the initial cohort population. [file Table_1.docx]

**Supplementary Table 1.** Detailed balance before and after propensity score adjustment in the initial cohort population.

| Covariates | Higher MALAT1 | | Lower MALAT1 | | Std. Mean Diff. | |
| --- | --- | --- | --- | --- | --- | --- |
|  | **Before** | **After** | **Before** | **After** | **Before** | **After** |
| Propensity (all cases) | 0.581 | 0.581 | 0.419 | 0.579 | 0.915 | 0.008 |
| Gender(male vs. female) | 0.512 | 0.512 | 0.622 | 0.492 | -0.218 | 0.039 |
| Age (yr) | 58.122 | 58.122 | 58.134 | 58.621 | -0.001 | -0.046 |
| Age_subgroup (≥60 vs. <60) | 0.463 | 0.463 | 0.439 | 0.457 | 0.049 | 0.012 |
| BMI (kg/m^2^) | 23.398 | 23.398 | 23.715 | 23.204 | -0.103 | 0.063 |
| BMI_subgroup (≥24 vs. <24) | 0.476 | 0.476 | 0.463 | 0.363 | 0.024 | 0.225 |
| Tumor Location |  |  |  |  |  |  |
| Left colon vs. Right colon | 0.305 | 0.305 | 0.232 | 0.256 | 0.158 | 0.106 |
| Rectum vs. Right colon | 0.585 | 0.585 | 0.646 | 0.610 | -0.123 | -0.050 |
| Tumor size (mm) | 61.000 | 61.000 | 53.646 | 57.986 | 0.212 | 0.087 |
| CEA (ng/mL) | 15.915 | 15.915 | 15.288 | 14.085 | 0.029 | 0.085 |
| CEA-Group (>5 vs. ≤5) | 0.537 | 0.537 | 0.451 | 0.455 | 0.170 | 0.163 |
| CA199 (U/mL) | 32.625 | 32.625 | 24.556 | 32.839 | 0.299 | -0.008 |
| CA199-Group (>37 vs. ≤37) | 0.341 | 0.341 | 0.171 | 0.291 | 0.358 | 0.107 |
| T-Stage |  |  |  |  |  |  |
| T2 vs. T1 | 0.037 | 0.037 | 0.122 | 0.031 | -0.452 | 0.031 |
| T3 vs. T1 | 0.171 | 0.171 | 0.256 | 0.158 | -0.225 | 0.034 |
| T4 vs. T1 | 0.720 | 0.720 | 0.561 | 0.755 | 0.351 | -0.077 |
| N-Stage |  |  |  |  |  |  |
| N1 vs. N0 | 0.329 | 0.329 | 0.463 | 0.297 | -0.284 | 0.067 |
| N2 vs. N0 | 0.061 | 0.061 | 0.049 | 0.044 | 0.051 | 0.072 |
| M-Stage (M1 vs. M0) | 0.061 | 0.061 | 0.134 | 0.107 | -0.304 | -0.192 |
| Histopathological Morphology (infiltrating ulcer vs. protruding ) | 0.341 | 0.341 | 0.268 | 0.386 | 0.153 | -0.093 |
| Differentiation  (high vs. low to medium) | 0.378 | 0.378 | 0.366 | 0.375 | 0.025 | 0.006 |
| Adjuvant Chemotherapy (yes vs. no) | 0.415 | 0.415 | 0.341 | 0.431 | 0.148 | -0.034 |
