# Supplementary material for: Long Noncoding RNA MALAT1 and Colorectal Cancer: A Propensity Score Analysis of Two Prospective Cohorts
Source: Front Oncol. 2022 Apr 26;12:824767. doi: 10.3389/fonc.2022.824767 (PMC9088002; doi:10.3389/fonc.2022.824767)
Supplement: Supplementary Table 2 — Detailed balance before and after propensity score adjustment in the external TCGA cohort population. [file Table_2.docx]

**Supplementary Table 2.** Detailed balance before and after propensity score adjustment in the external TCGA cohort population.

| Covariates | Higher MALAT1 | | Lower MALAT1 | | Std. Mean Diff. | |
| --- | --- | --- | --- | --- | --- | --- |
|  | **Before** | **After** | **Before** | **After** | **Before** | **After** |
| Propensity (all cases) | 0.523 | 0.523 | 0.477 | 0.523 | 0.461 | 0.001 |
| Gender(male vs. female) | 0.460 | 0.460 | 0.460 | 0.474 | 0.000 | -0.029 |
| Age (yr) | 66.940 | 66.940 | 65.117 | 66.956 | 0.155 | -0.001 |
| Age_subgroup (≥60 vs. <60) | 0.725 | 0.725 | 0.691 | 0.713 | 0.075 | 0.027 |
| BMI (kg/m^2^) | 28.642 | 28.642 | 27.273 | 28.427 | 0.237 | 0.037 |
| BMI_ subgroup (≥25 vs. <25) | 0.735 | 0.735 | 0.611 | 0.723 | 0.281 | 0.028 |
| Tumor Location |  |  |  |  |  |  |
| Left colon vs. Right colon | 0.383 | 0.383 | 0.372 | 0.378 | 0.021 | 0.009 |
| Rectum vs. Right colon | 0.215 | 0.215 | 0.191 | 0.219 | 0.057 | -0.010 |
| History of Polyps (yes vs. no) | 0.346 | 0.346 | 0.289 | 0.380 | 0.120 | -0.072 |
| CEA (ng/mL) | 18.582 | 18.582 | 17.183 | 20.536 | 0.054 | -0.076 |
| CEA-Group (>5 vs. ≤5) | 0.597 | 0.597 | 0.520 | 0.606 | 0.157 | -0.019 |
| T-Stage |  |  |  |  |  |  |
| T2 vs. T1 | 0.171 | 0.171 | 0.174 | 0.187 | -0.009 | -0.042 |
| T3 vs. T1 | 0.681 | 0.681 | 0.691 | 0.641 | -0.022 | 0.086 |
| T4 vs. T1 | 0.114 | 0.114 | 0.101 | 0.139 | 0.042 | -0.079 |
| N-Stage |  |  |  |  |  |  |
| N1 vs. N0 | 0.242 | 0.242 | 0.248 | 0.248 | -0.016 | -0.015 |
| N2 vs. N0 | 0.205 | 0.205 | 0.171 | 0.185 | 0.083 | 0.048 |
| M-Stage (M1 vs. M0) | 0.178 | 0.178 | 0.121 | 0.159 | 0.149 | 0.050 |
