# Supplementary material for: Long Noncoding RNA MALAT1 and Colorectal Cancer: A Propensity Score Analysis of Two Prospective Cohorts
Source: Front Oncol. 2022 Apr 26;12:824767. doi: 10.3389/fonc.2022.824767 (PMC9088002; doi:10.3389/fonc.2022.824767)
Supplement: Supplementary Table 3 — Sensitivity analysis by using confounding RR analysis. [file Table_3.docx]

**Supplementary Table 3.** Sensitivity analysis by using confounding RR analysis.

| Populations | Models | HRs and 95% CIs for OS | Confounding RR (P-value) | HRs and 95% CIs for DFS | Confounding RR (P-value) |
| --- | --- | --- | --- | --- | --- |
| Initial cohort | Univariate | 1.428 (0.901-2.263) | 0.761 (0.181) | 1.525 (0.975-2.387) | 0.754 (0.082) |
|  | PS-adjustment | 1.087 (0.657-1.797) |  | 1.150 (0.710-1.865) |  |
| External cohort | Univariate | 1.072 (0.750-1.532) | 0.906 (0.873) | 1.266 (0.948-1.690) | 0.930 (0.057) |
|  | PS-adjustment | 0.971 (0.676-1.396) |  | 1.177 (0.878-1.577) |  |
| Combined populations | Univariate | 1.194 (0.901-1.583) | 0.846 (0.349) | 1.337 (1.049-1.704) | 0.875 (0.011) |
|  | PS-adjustment | 1.010 (0.752-1.355) |  | 1.170 (0.910-1.502) |  |
