# Supplementary material for: Long Noncoding RNA MALAT1 and Colorectal Cancer: A Propensity Score Analysis of Two Prospective Cohorts
Source: Front Oncol. 2022 Apr 26;12:824767. doi: 10.3389/fonc.2022.824767 (PMC9088002; doi:10.3389/fonc.2022.824767)
Supplement: Supplementary Table 4 — Sensitivity analysis by using of conventional multivariate Cox hazard regression method. [file Table_4.docx]

**Supplementary Table 4.** Sensitivity analysis by using of conventional multivariate Cox hazard regression method.

| Populations | Multivariate adjusted HR and 95% CI, P-value | |
| --- | --- | --- |
|  | **OS** | **DFS** |
| Initial cohort^a^ | 1.037 (0.624-1.725), 0.888 | 1.261 (0.764-2.079), 0.364 |
| External cohort^b^ | 0.974 (0.674-1.408), 0.890 | 1.184 (0.883-1.587), 0.260 |
| Combined populations | 0.995 (0.739-1.341), 0.975 | 1.203 (0.934-1.550), 0.152 |

^a^**Initial cohort**: OS anlaysis, adjusted for gender, age, BMI, AJCC stage, CA 19-9, adjuvant chemotherapy. DFS anslysis, adjusted for gender, age, BMI, AJCC stage, CA 19-9, CEA, tumor location, and adjuvant chemotherapy.
^b^**External cohort**: OS analysis, adjusted for gender, age, BMI, tumor location, T stage, N stage, M stage, and CEA. DFS analysis, adjusted fro gender, age, BMI, T stage, N stage, M stage, and CEA.
