# Supplementary material for: Long Noncoding RNA MALAT1 and Colorectal Cancer: A Propensity Score Analysis of Two Prospective Cohorts
Source: Front Oncol. 2022 Apr 26;12:824767. doi: 10.3389/fonc.2022.824767 (PMC9088002; doi:10.3389/fonc.2022.824767)
Supplement: Supplementary Table 5 — Sensitivity analysis by excluding patients with shorter follow-up duration. [file Table_5.docx]

**Supplementary Table 5.** Sensitivity analysis by excluding patients with shorter follow-up duration.

| Sensitivity Analysis | Models | HR (95% CI), P-value | |
| --- | --- | --- | --- |
|  |  | **OS** | **DFS** |
| Excluding patients  with follow-up period ≤ 1 mo. | Univariate | 1.055 (0.737-1.509), 0.770 | 1.254 (0.939-1.675), 0.126 |
|  | PS-adjustment | 0.954 (0.663-1.373), 0.801 | 1.164 (0.868-1.561), 0.310 |
| Excluding patients  with follow-up period ≤ 3 mo. | Univariate | 0.933 (0.640-1.358), 0.716 | 1.174 (0.872-1.581), 0.290 |
|  | PS-adjustment | 0.856 (0.584-1.254), 0.424 | 1.101 (0.815-1.489), 0.530 |
