# Supplementary material for: Long Noncoding RNA MALAT1 and Colorectal Cancer: A Propensity Score Analysis of Two Prospective Cohorts
Source: Front Oncol. 2022 Apr 26;12:824767. doi: 10.3389/fonc.2022.824767 (PMC9088002; doi:10.3389/fonc.2022.824767)
Supplement: Supplementary Table 6 — Associations between MALAT1 expression level and clinical/pathological characteristics in the initial cohort population. [file Table_6.docx]

**Supplementary Table 6. A**ssociations between MALAT1 expression level and clinical/pathological characteristics in the initial cohort population.

| Factors | MALAT1  Expression Level | | Univariate Model | | Multivariate Model | |
| --- | --- | --- | --- | --- | --- | --- |
|  | **Lower** | **Higher** | **OR (95% CI)** | **P-value** | **OR (95% CI)** | **P-value** |
| Gender |  |  |  |  |  |  |
| Female | 51 | 42 | - | - | - | - |
| Male | 31 | 40 | 1.567 (0.841-2.918) | 0.157 | 1.525 (0.797-2.917) | 0.202 |
| Age (years) |  |  |  |  |  |  |
| < 60 | 46 | 44 | - | - | - | - |
| ≥ 60 | 36 | 38 | 1.104 (0.596-2.042) | 0.754 | 1.090 (0.571-2.080) | 0.795 |
| BMI (kg/m^2^) |  |  |  |  |  |  |
| < 24 | 44 | 43 | - | - | - | - |
| ≥ 24 | 38 | 39 | 1.050 (0.569-1.939) | 0.876 | 1.108 (0.583-2.105) | 0.755 |
| Location site |  |  |  |  |  |  |
| Right Colon | 10 | 9 | - | - | - | - |
| Left Colon | 19 | 25 | 1.462 (0.496-4.306) | 0.491 |  |  |
| Rectum | 53 | 48 | 1.006 (0.377-2.685) | 0.990 |  |  |
| History of cancers |  |  |  |  |  |  |
| No | 69 | 73 | - | - |  |  |
| Yes | 13 | 9 | 0.654 (0.263-1.628) | 0.362 |  |  |
| Tumor Size (diameter, mm) |  |  |  |  |  |  |
| ≤ 40 | 31 | 27 | - | - |  |  |
| > 40 | 51 | 55 | 1.238 (0.652-2.351) | 0.514 |  |  |
| CEA (ng/mL) |  |  |  |  |  |  |
| ≤ 5 | 45 | 38 | - | - |  |  |
| > 5 | 37 | 44 | 1.408 (0.762-2.603) | 0.275 |  |  |
| CA19-9 (U/mL) |  |  |  |  |  |  |
| ≤ 37 | 68 | 54 | - | - | - | - |
| > 37 | 14 | 28 | **2.519 (1.208-5.249)** | **0.014** | **2.522 (1.188-5.355)** | **0.016** |
| T-stage |  |  |  |  |  |  |
| T1-3 | 36 | 23 | - | - | - | - |
| T4 | 46 | 59 | **2.008 (1.048-3.845)** | **0.036** | **2.094 (1.072-4.090)** | **0.030** |
| N-stage |  |  |  |  |  |  |
| N0 | 40 | 50 | - | - |  |  |
| N1 or N2 | 42 | 32 | 0.610 (0.328-1.133) | 0.118 |  |  |
| M-stage |  |  |  |  |  |  |
| M0 | 71 | 77 | - | - |  |  |
| M1 | 11 | 5 | 0.419 (0.139-1.266) | 0.123 |  |  |
| Histopathological Morphology | |  |  |  |  |  |
| Protruding | 60 | 54 | - | - |  |  |
| Infiltrating ulcer | 22 | 28 | 1.414 (0.715-2.760) | 0.310 |  |  |
| Differentiation |  |  |  |  |  |  |
| Low to Medium | 52 | 51 | - | - |  |  |
| High | 30 | 31 | 1.054 (0.559-1.985) | 0.872 |  |  |
