# Supplementary material for: Long Noncoding RNA MALAT1 and Colorectal Cancer: A Propensity Score Analysis of Two Prospective Cohorts
Source: Front Oncol. 2022 Apr 26;12:824767. doi: 10.3389/fonc.2022.824767 (PMC9088002; doi:10.3389/fonc.2022.824767)
Supplement: Supplementary Table 7 — Associations between MALAT1 expression level and clinical/pathological characteristics in the external cohort population. [file Table_7.docx]

**Supplementary Table 7.** Associations between MALAT1 expression level and clinical/pathological characteristics in the external cohort population.

| Factors | MALAT1 Expression Level | | Univariate Model | | Multivariate Model | |
| --- | --- | --- | --- | --- | --- | --- |
|  | **Lower** | **Higher** | **OR (95% CI)** | **P-value** | **OR (95% CI)** | **P-value** |
| Gender |  |  |  |  |  |  |
| Female | 137 | 137 | - | - | - | - |
| Male | 161 | 161 | 1.000 (0.725-1.380) | 1.000 | 0.952 (0.683-1.326) | 0.770 |
| Age (years) |  |  |  |  |  |  |
| < 60 | 92 | 82 | - | - | - | - |
| ≥ 60 | 206 | 216 | 1.176 (0.826-1.675) | 0.368 | 1.218 (0.844-1.760) | 0.292 |
| BMI (kg/m^2^) |  |  |  |  |  |  |
| < 25 | 116 | 79 | - | - | - | - |
| ≥ 25 | 182 | 219 | **1.767 (1.249-2.500)** | **0.001** | **1.876 (1.313-2.679)** | **0.001** |
| Location site |  |  |  |  |  |  |
| Right Colon | 130 | 120 | - | - |  |  |
| Left Colon | 111 | 114 | 1.113 (0.776-1.595) | 0.562 |  |  |
| Rectum | 57 | 64 | 1.216 (0.788-1.879) | 0.377 |  |  |
| History of polyps |  |  |  |  |  |  |
| No | 212 | 195 | - | - | - | - |
| Yes | 86 | 103 | 1.302 (0.921-1.841) | 0.135 | 1.347 (0.941-1.928) | 0.103 |
| CEA (ng/mL) |  |  |  |  |  |  |
| ≤ 5 | 143 | 120 | - | - | - | - |
| > 5 | 155 | 178 | 1.368 (0.989-1.893) | 0.058 | 1.356 (0.965-1.907) | 0.080 |
| T-stage |  |  |  |  |  |  |
| T1-3 | 268 | 264 | - | - |  |  |
| T4 | 30 | 34 | 1.151 (0.684-1.934) | 0.597 |  |  |
| N-stage |  |  |  |  |  |  |
| N0 | 173 | 165 | - | - |  |  |
| N1 or N2 | 125 | 133 | 1.116 (0.807-1.543) | 0.508 |  |  |
| M-stage |  |  |  |  |  |  |
| M0 | 262 | 245 | - | - | - | - |
| M1 | 36 | 53 | 1.574 (0.996-2.488) | 0.052 | 1.399 (0.865-2.264) | 0.171 |
