# Supplementary material for: Long Noncoding RNA MALAT1 and Colorectal Cancer: A Propensity Score Analysis of Two Prospective Cohorts
Source: Front Oncol. 2022 Apr 26;12:824767. doi: 10.3389/fonc.2022.824767 (PMC9088002; doi:10.3389/fonc.2022.824767)
Supplement: Supplementary Table 8 — E-value analyses for the pooled effect estimates of the meta-analysis. [file Table_8.docx]

**Supplementary Table 8.** E-value analyses for the pooled effect estimates of the meta-analysis.

| Outcome | Pooled HR (95% CI), P-value | E-values for^a^ | |
| --- | --- | --- | --- |
|  |  | **HR Point Estimate** | **HR Lower CI Limit** |
| OS | 1.683 (0.917-3.087), 0.093 | 2.221 | 1.000 |
| DFS | 1.784 (1.021-3.118) , 0.042 | 2.347 | 1.136 |

^a^E-value shows the minimum strength of association that a hypothetical residual confounding factor would need to be associated with both the expression status of MALAT1 and the outcomes of CRC patients, and at the same time, the hypothetical confounding factor must be frequent to be able to fully explain the observed association between MALAT1 expression and CRC outcomes.
